# Ovatodiolide of* Anisomeles indica* Exerts the Anticancer Potential on Pancreatic Cancer Cell Lines through STAT3 and NF-*κ*B Regulation

**DOI:** 10.1155/2016/8680372

**Published:** 2016-05-08

**Authors:** Ya-Ju Hsieh, Sung-Pin Tseng, Yu-Hsuan Kuo, Tain-Lu Cheng, Chiao-Yu Chiang, Yew-Min Tzeng, Wan-Chi Tsai

**Affiliations:** ^1^Department of Medical Imaging and Radiological Sciences, Kaohsiung Medical University, Kaohsiung 80708, Taiwan; ^2^Department of Medical Laboratory Science and Biotechnology, Kaohsiung Medical University, Kaohsiung 80708, Taiwan; ^3^Department of Laboratory Medicine, Kaohsiung Medical University Hospital, Kaohsiung, Taiwan; ^4^Department of Hemato-Oncology, Chi-Mei Medical Center, Tainan 71004, Taiwan; ^5^Department of Biomedical Science and Environmental Biology, Center for Biomarkers and Biotech Drugs, Kaohsiung Medical University, Kaohsiung 80708, Taiwan; ^6^Center for General Education, National Taitung University, Taitung 95092, Taiwan; ^7^Department of Life Science, National Taitung University, Taitung 95092, Taiwan; ^8^Institute of Biochemical Sciences and Technology, Chaoyang University of Technology, Taichung 41349, Taiwan; ^9^Center for Infectious Disease and Cancer Research, Kaohsiung Medical University, Kaohsiung 80708, Taiwan

## Abstract

Pancreatic cancer is the eighth leading cause of cancer death worldwide. Patients with pancreatic cancer are normally diagnosed at an advanced stage and present poor survival rate. Ovatodiolide (OV), a bioactive macrocyclic diterpenoid isolated from* Anisomeles indica*, showed cytotoxicity effects in pancreatic cancer cells by inhibiting cell proliferation and inducing apoptosis. Moreover, not only were cell adhesion and invasion markedly suppressed in a dose-dependent manner, but the mRNA expression of matrix metalloproteinase-9 (MMP-9) and focal adhesion kinase (FAK) was also significantly decreased. Western blot analysis indicated that OV potently suppressed the phosphorylation of STAT-3 and its upstream kinase including ERK1/2, P38, and AKT Ser473. Meanwhile, OV inactivated the nuclear factor kappa B (NF-*κ*B) by inhibiting I*κ*B kinase (IKK *α*/*β*) activation and the subsequent suppression of inhibitor of kappa B (I*κ*B) phosphorylation. These results demonstrated that OV could potentially inhibit Mia-PaCa2 cancer cells proliferation and induce apoptosis through modulation of NF-*κ*B and STAT3 pathway. Moreover, OV suppressed cell invasiveness and interfered with cell-matrix adhesion in Mia-PaCa2 cancer cells by reducing MMP-9 and FAK transcription through suppressing NF-*κ*B and STAT3 pathway. Taken together, our findings reveal a new therapeutic and antimetastatic potential of ovatodiolide for pancreatic cancer remedy.

## 1. Introduction

Pancreatic cancer is the eighth leading cause of cancer death in men and the ninth in women worldwide [1]. The treatment options mainly depend on the stage of the cancer. Once patient has been diagnosed as having pancreatic cancer, they should be evaluated for possible surgical therapy since this provides the best option for long-term survival. Unfortunately, the symptoms do not usually appear in the disease's early stages and are individually not distinctive to the disease [2]. Therefore, pancreatic cancer (85% of which is adenocarcinoma) is normally diagnosed at an advanced stage. By that time tumor often spreads to nearby blood vessels and organs that make it unresectable. This leads to the result that only 15–20% of patients with pancreatic cancer are able to have surgery and the 5-year survival rate is only around 5% [3].

Gemcitabine, approved by Food and Drug Administration (FDA) in 1997, has been used to treat cancer patients before (neoadjuvant) or after surgery (adjuvant treatment). For those who have advanced and unresectable cancers, gemcitabine could be given alone or with radiation therapy (chemoradiation). However, it has been reported that pancreatic cancer was intrinsically resistant to 5-fluorouracil (FU) and an acquired resistance to gemcitabine was rapidly developed after continuous exposure to this drug [4]. Therefore, tumor recurrence usually occurs and few of the patients achieve long-term survival [5]. Recently, some chemotherapy regimens using FOLFIRINOX (fluorouracil [5-FU], leucovorin, irinotecan, and oxaliplatin) or a combination of gemcitabine with albumin-bound-paclitaxel (nab-paclitaxel) show a benefit over gemcitabine in metastatic pancreatic cancer [6, 7]. But the development of these treatments is limited by the substantial side effects and is thus only suitable for people who are able to tolerate the side effects. These clinical observations prompted us to investigate a potential drug as alternative option for treating pancreatic cancer.

*Anisomeles indica* (L.) Kuntze (Labiatae), a camphor-scented perennial woody shrub, is commonly used in traditional medicines for the treatment of inflammatory skin disease, gastric dysfunction, hepatic disorders, and immune system deficiencies [8]. It was also reported that both the whole* A. indica* extracts and the purified methanolic extract, ovatodiolide (a bioactive cembrane-type diterpenoid), exerted anti-inflammatory activity through inhibiting NO, TNF-*α*, and IL-12 in a dose-dependent manner [9, 10]. In addition, these extracts induced cell cycle arrest at the G0/G1 phase in both mitogen-stimulated spleen cells and tumor cells [9]. In human oral squamous cell carcinoma, OV also inhibited cell proliferative activity through inducing G2/M arrest, apoptosis, and disturbance of intracellular redox balance. [11].

Recently, OV has been shown to inhibit migration and invasion of MDA-MB-231 and MCF-7 breast cancer cell lines by a decreased matrix metallopeptidase 9 activity, revealing the repression role of OV in tumor metastasis [12, 13]. However, the efficacy of OV to treat chemoresistant pancreatic cancer remains largely unknown. Thus, this study aims to understand whether OV possesses potent cytotoxic effect against pancreatic cancer cells and to further decipher the possible mechanisms.

## 2. Materials and Methods

### 2.1. Cell Culture

The human pancreatic cancer cell lines Mia-PaCa2 (BCRC 60139) and PANC-1 (BCRC 60284) were purchased from Bioresource Collection and Research Center in Taiwan. Both cell lines were cultured in RPMI-1640 medium (Gibco-Invitrogen) supplemented with L-glutamine (2 mM), sodium pyruvate (1 mM), penicillin (100 U/mL), streptomycin (100 mg/mL), and 10% fetal bovine serum. The HPDE-E6E7 pancreatic duct epithelial cell line, a generous gift from Dr. Ming-Sound Tsao (Ontario Cancer Institute, Toronto, ON, Canada) [14], was cultured in keratinocyte serum-free medium supplemented with epidermal growth factor and bovine pituitary extract (Invitrogen #17005-042). Cells were incubated in a humidified incubator at 37°C and 5% CO_2_/95% air.

### 2.2. MTT (3-[4,5-Dimethylthiazol-2-yl]-2,5-diphenyltetrazolium Bromide) Cell Viability Assay

Cells were plated in 96-well plates with 5,000 cells per well. Following the overnight incubation cells were treated with 2.5, 5, 10, or 20 *μ*M of OV for 24 h. The DMSO-treated cells served as control. To quantify the cell viability, the medium was replaced by 150 *μ*L of medium containing 10% MTT (Sigma-Aldrich) solution. After 1 h of incubation at 37°C, the MTT solution in wells was removed and the formazan crystals within cells were solubilized by 100 *μ*L of DMSO. The absorbance of each sample at 595 nm was measured by an enzyme-linked immunosorbent assay reader (Power Wave 340; Bio-Tek Instruments, Inc.)/microplate reader (Bio-Rad Laboratories, Richmond, CA, USA). Cell viability was determined by normalizing the absorbance value of each sample by that of DMSO-treated control cells and represented as mean ± SD of four independent experiments performed in triplicate.

### 2.3. BrdU Cell Proliferation Assay

The antiproliferative effect of OV in Mia-PaCa2 pancreatic cancer cells was evaluated by using Cell Proliferation ELISA, BrdU (colorimetric) Kit (Roche Applied Science, Indianapolis, IN). Briefly, the cells cultured in 96-well plates (5000 cells/100 *μ*L/well) were treated with 2.5, 5, 10, or 20 *μ*M of OV for 24 h. After drug treatment, cells in each well were labeled with 10 *μ*M of BrdU for 4 h at 37°C. Then the medium was removed and FixDenat reagent (100 *μ*L/well) was used to fix cells and denature the DNA. The cells were further incubated with the anti-BrdU-POD antibody (1 : 100) for 90 min at room temperature. Following the washing step, substrate reaction was performed and the reaction product was quantified by measuring the absorbance using a scanning multiwell spectrophotometer (ELISA reader) at 370 nm.

### 2.4. Flow Cytometric Analysis of Cell Apoptosis

The measurement of phosphatidylserine (PS) on the outer leaflet of the plasma membrane in apoptotic cells was conducted according to the manufacturer's instructions for the Alexa Fluor 488 Annexin V/Dead Cell Apoptosis Kit (Invitrogen). Propidium iodide (PI) was used to stain the nucleic acids in the dead cell with red fluorescence. After 24 h treatment of OV or DMSO (control), Mia PaCa-2 pancreatic cancer cells were harvested and resuspended in 100 *μ*L Annexin-binding buffer containing 5 *μ*L of Alexa Fluor 488 Annexin V and 1 *μ*L of PI (100 *μ*g/mL) for 15 min at room temperature. Subsequently, each reaction was added with 400 *μ*L of Annexin-binding buffer and then cells were analyzed by flow cytometry (Becton, Dickinson and Company, Palo Alto, CA, USA). Cells that showed green fluorescence were early apoptotic (Annexin V-FITC+/PI−) while those that showed red and green fluorescence were classified as late apoptotic or necrotic (Annexin V-FITC+/PI+) cells.

### 2.5. Cell Adhesion Assay

Mia-PaCa2 pancreatic cancer cells were grown to approximately 80% confluence in RPMI-1640 containing 10% FBS and trypsinized, and then the cells were seeded at 1 × 10^4^ concentration in RPMI-1640 medium containing 0, 2.5, 5, 10, or 20 *μ*M of OV. The cells were allowed to adhere to the bottom of the wells for 2 h in a 37°C CO_2_ incubator. After vigorously shaking on the shaker for 15 sec, subsequently the unbound cells were removed from the wells and washed out by PBS. The attached cells were followed by MTT assay to measure the cell population. One hundred microliters of MTT staining solution was added to each well. After 1 h, the cells were lysed with DMSO and read at A595.

### 2.6. Matrigel Invasion Assay

Mia-PaCa2 invasiveness was studied using Matrigel invasion chambers (BD Bio, Germany), according to the manufacturer's instructions. In brief, Matrigel at a concentration of 1 mg/mL was added into transwell insert and incubated overnight at 37°C for solidation. The cells (2 × 10^4^) mixed with serum-free medium containing DMSO or OV were plated in the upper chamber, and 500 *μ*L RPMI-1640 medium containing 10% FBS was added to the bottom chamber. After 37°C incubation for 24 h, the uninvaded cells were removed from the upper chamber with a cotton swab. Invaded cells adhered to the insert membranes were fixed with formaldehyde (4%) for 30 min and permeabilized with triton X-100 (0.2%) for 10 min, followed by DAPI staining (5 *μ*g/mL). The number of invaded cells was counted in 5 random fields (40x) under the fluorescent microscopy.

### 2.7. Western Blot Analysis

OV- or DMSO-treated Mia-PaCa2 pancreatic cancer cells were lyzed in RIPA lysis buffer (Cell Signaling Technology, Beverly, MA, USA) containing 1% of protease inhibitor for 5 min on ice and then subjected to sonication for 20 sec. The BCA Protein Assay kit (Pierce Biotechnology, Inc., Rockford, IL, USA) was used to measure protein concentration. Equal amounts of protein were separated using sodium dodecyl sulfate-polyacrylamide gel electrophoresis (SDS-PAGE) and transferred to a nitrocellulose (NC) or polyvinylidene difluoride (PVDF) membrane. After blocking with 5% of bovine serum albumin (BSA), primary antibodies against STAT-3, phospho-STAT-3 Tyr705, phospho-STAT-3 Ser705, AKT, phospho-AKT Ser473, phospho-AKT Thr308, ERK 1/2, phospho-ERK 1/2 Thr202/Tyr204, P38, phospho-P38 Thr180/Tyr182, or *β*-actin were applied at 4°C overnight, followed by horseradish peroxidase-conjugated secondary antibody for 1 h at room temperature. Protein band was detected using ECL Western Blotting Detection System (GE Healthcare, Buckinghamshire, UK). The *β*-actin was served as loading control.

### 2.8. RNA Extraction and Quantitative Real-Time PCR (Q-PCR)

Total RNA was extracted from OV- or DMSO-treated Mia-PaCa2 pancreatic cancer cells by Trizol reagent (Invitrogen). One *μ*g of total RNA was reversely transcribed into cDNA following the manufacturer's protocol (High-Capacity cDNA Reverse Transcription Kit; Applied Biosystems, Foster City, CA). For real-time PCR analysis, all reactions were prepared by mixing 50 ng of cDNA with FastStart Universal SYBR Green Master (ROX) (Roche) and corresponding primers. The primer sequences were designed on Universal Probe Library Assay Design Center (Roche Applied Science; http://www.roche-applied-science.com/sis/rtpcr/upl/ezhome.html). The primer sequences were as follows: matrix metallopeptidase 9 (MMP-9; Forward: 5′-GTGGCAGGGGGAAGATGC-3′; Reverse: 5′-TCAGGGCACTGCAGGATG-3′), focal adhesion kinase (FAK; Forward: 5′-CCCTGCTGACAGCTACAACG-3′; Reverse: 5′-GCCCGTCACATTCTCGTACA-3′), and *β*-actin (Forward: 5′-TTCTACAATGAGCTGCGTGTG-3′; Reverse: 5′-ATCACAATGCCAGTGGTACG-3′). Real-time PCR was performed in the Applied Biosystems 7500 system with the following program: 95°C for 10 min followed by 40 cycles of denaturation at 95°C for 10 sec and amplification at 60°C for 1 min. All samples were prepared in duplicate and the threshold cycle (Ct) values detected from each sample were normalized to that of its *β*-actin RNA.

## 3. Results

### 3.1. OV Had Selective Cytotoxicity on Pancreatic Cancer Cells

**Figure 1:**
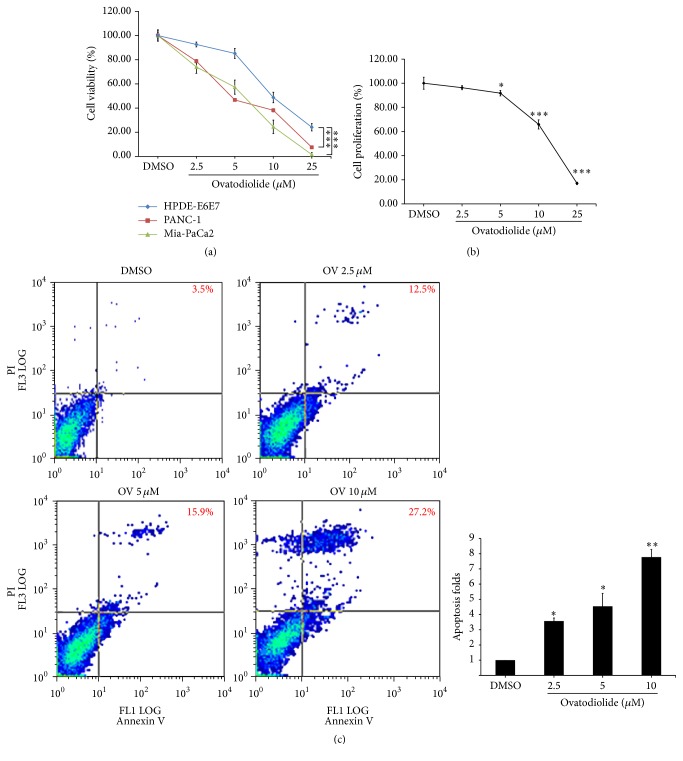
Ovatodiolide could decrease the cell viability by inducing apoptosis and inhibiting cell proliferation on pancreatic cancer cells. (a) OV was treated at indicated concentration for 24 h; then the cell viability and BrdU incorporation were measured by MTT assay and Cell Proliferation ELISA, BrdU (colorimetric) Kit, respectively. The survival rates were significantly decreased on 2 pancreatic cancer cell lines, Panc-1 and Mia-PaCa2. (b) The cell proliferation rate of Mia-PaCa2 declined gradually upon OV treatment. Each point represented the mean ± SD (*n* = 6). ^*∗*^ *P* < 0.05, ^*∗∗*^ *P* < 0.01, and ^*∗∗∗*^ *P* < 0.001. (c) The Annexin V/PI staining indicated the population of cell death, including apoptosis. Mia-PaCa2 cells were treated with OV at 2.5, 5, and 10 *μ*M for 24 h. The Alexa Fluor® 488 Annexin V/Dead Cell Apoptosis Assay was used to conduct the experiment. The Annexin V-FITC signal is shown on the *x*-axis and PI signal is shown on the *y*-axis. The number showed in upper-right corner represented the sum of cell population in 1st and 4th quadrant.

To evaluate the cytotoxic effect of OV, pancreatic cancer cells (PANC-1 and Mia-PaCa2) and pancreatic duct epithelial cells (HPDE-E6E7) were treated with DMSO or various concentrations of OV for 24 h, and cell viability was analyzed by MTT assay. As shown in Figure 1(a), exposure to OV resulted in a dose-dependent decrease of cell viability in all cell lines. However, the cytotoxic effects of OV in pancreatic cancer cells were significantly greater than that in pancreatic duct epithelial cells (*P* < 0.001), with the IC_50_ value of OV being 4.14, 6.11, and 9.83 *μ*M in PANC-1, Mia-PaCa2, and HPDE-E6E7 cells, respectively. As Mia-PaCa2 showed higher aggressive phenotype and tumorigenic potential than PANC-1 [15–17], it was used for following experiments.

### 3.2. OV Could Inhibit Pancreatic Cancer Cells Proliferation and Induce Apoptosis

We further investigated whether the cytotoxic effect of OV was mediated by inhibition of cell proliferation and/or apoptosis. After OV treatment for 24 h, Mia-PaCa2 cells were labeled with BrdU for the quantification of cell proliferation. OV at 2.5, 5, 10, and 25 *μ*M resulted in 96.2, 91.7, 65.8, and 17% of cell proliferation, respectively, while comparing with DMSO-treated control cells (Figure 1(b)). The data demonstrated the antiproliferation effect of OV. By using flow cytometric analysis to evaluate the OV-induced apoptosis, the sum of cells that showed Annexin V-FITC+/PI− (early-stage apoptosis) and those that showed Annexin V-FITC+/PI+ (late-stage apoptosis) were defined as total apoptotic events. As shown in Figure 1(c), 24 h treatment of OV significantly increases the percentage of apoptotic Mia-PaCa2 cells in a dose-dependent manner. The fold increases in apoptotic events at 2.5, 5, and 10 *μ*M of OV were 3.6, 4.5, and 7.8, respectively, as compared to DMSO control. These results suggested that cytotoxicity of OV on Mia-PaCa2 cells was contributed by inhibition of cell proliferation and induction of apoptosis.

### 3.3. OV Could Diminish the Invasion and Adhesion Ability of Mia-PaCa2 Cells

**Figure 2:**
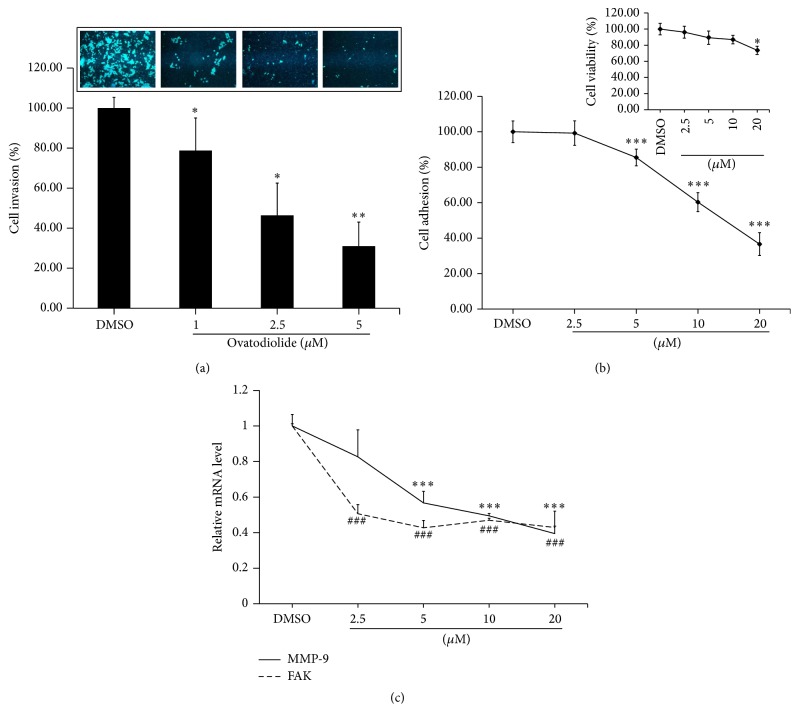
Ovatodiolide could inhibit Mia-PaCa2 invasion and adhesion ability. (a) 2 × 10^4^ Mia-PaCa2 cells were seeded on Matrigel-coated insert with indicated concentration of OV and then incubated in indicated concentration of OV-containing medium for 24 h. The cells on opposite site of membrane were stained with DAPI for cell number counting. The upper photos showed the invaded cells under fluorescent microscopy, and the lower bar graph represented the mean ± SEM (*n* = 5). The experiments were repeated 3 times. (b) 1 × 10^4^ Mia-PaCa2 cells in OV-containing medium were seeded in 96-well plate for 2 h; the attached cells were stained by MTT. The percentage of attached cells was shown in *x*-axis when the percentage of DMSO control group was normalized to 100. The adhesion ability of Mia-PaCa2 cells was inhibited by OV in a dose-dependent manner while the dosage was not harmful to the cells (upper-right). Each point represented the mean ± SD (*n* = 6), and the assay was repeated 3 times. (c) mRNA expression of MMP-9 and FAK was significantly decreased by OV after 24 h treatment. mRNA expressions were examined by real-time PCR and were normalized with the reference gene GAPDH. The data represented the mean ± SD (*n* = 6), and the assay was repeated 3 times. ^*∗*^ *P* < 0.05, ^*∗∗*^ *P* < 0.01, and ^*∗∗∗*^ *P* < 0.001. ^###^ represents the decrease of FAK expression after OV treatment is as significant as *P* < 0.001.

Given that invasion of cancer cells is an essential step for tumor metastasis and cell adhesion ability could help tumor cells colonize at new sites during metastasis [18–20], we further investigated whether OV had any impact on cell invasion and adhesion. As shown in Figure 2(a), the invasiveness of Mia-PaCa2 cells was dramatically suppressed by OV. Such reduction was dose-dependent with a 21 ± 0.09, 53 ± 0.07, and 69 ± 0.06% decrease when cells were treated with 1, 2.5, and 5 *μ*M of OV, respectively. For evaluating cell adhesion ability, we performed a short-term incubation (2 h) of Mia-PaCa2 cells with DMSO or OV at 37°C and then carried out analysis by MTT assay. Obviously, OV decreased Mia-PaCa2 adhesion ability with increasing doses (Figure 2(b)). As comparing with DMSO treatment, 2.5, 5, 10, and 20 *μ*M OV inhibited 1.5 ± 4.79, 16.1 ± 2.31, 44.3 ± 2.98, and 73.1 ± 4.46% of cell adhesion, respectively. To confirm that such finding was not due to OV-induced cell death, we analyzed the cell viability after 2 h treatment of OV by MTT assay. There was no significant difference in cell viability between DMSO-treated and OV-treated cells unless the dose of OV increased to 20 *μ*M (*P* < 0.05). Furthermore, the representative molecules of invasion and adhesion were also examined. After 24 h treatment of DMSO or OV, total RNA was extracted and reverse transcribed into cDNA for Q-PCR. A significant decrease in mRNA expression level of mmp-9 was observed in OV-treated Mia-PaCa2 cells in a dose-dependent manner (Figure 2(c)), whereas FAK expression was significantly suppressed even in low dose. These results suggested that OV possessed an antimetastatic potential through inhibiting invasion and adhesion of pancreatic cancer cells.

### 3.4. Inhibitory Effects of OV on STAT-3 and NF-*κ*B Cascade

**Figure 3:**
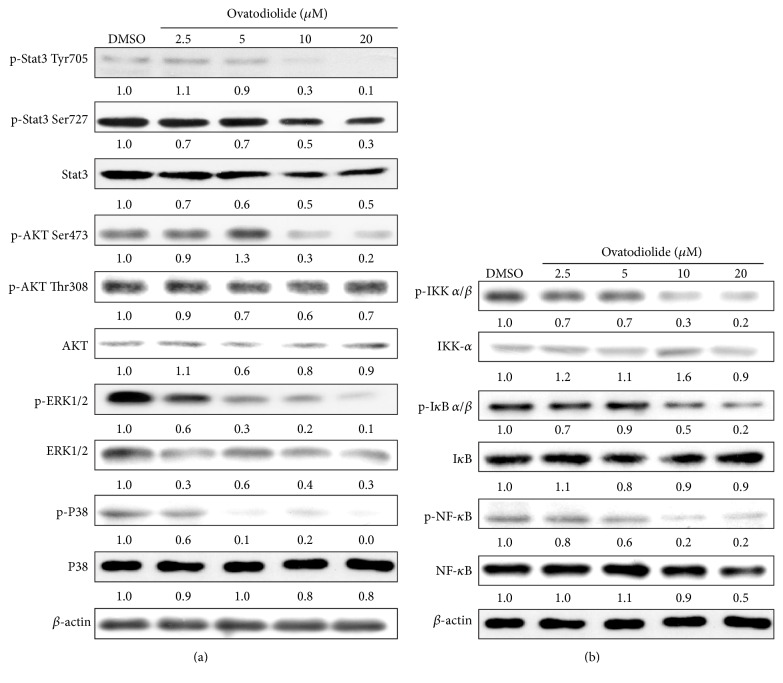
Dose-dependent effects of OV on the phosphorylation of STAT3, AKT, ERK, P38, and NF-*κ*B cascade. Cells were treated with the indicated concentrations of OV in 5% FBS-supplemented RPMI-1640 for 24 h, and cell lysates were immunoblotted as described in Section 2. The values in fold denote the relative intensity of protein bands of OV-treated samples to that of the respective DMSO vehicle control after being normalized to the respective internal control (*β*-actin).

As STAT-3 plays an important role in cell proliferation, apoptosis, invasion, adhesion, and regulation of FAK expression [21, 22], we performed Western blot to examine the effects of OV on STAT-3 and its activating kinase expression. As shown in Figure 3(a), OV markedly suppressed the phosphorylation of STAT-3 (Try705, Ser727). Such inhibitory effect of OV on STAT-3 was in parallel with a dephosphorylation of its upstream kinase including ERK1/2, P38, and AKT Ser473. In contrast, the phosphorylation of AKT Thr308 was not affected by OV. Interestingly, the total protein of STAT-3 and ERK1/2 was reduced after OV treatment as well, whereas the protein level of AKT and P38 did not change. These results indicated that the antimetastatic activity of OV might, at least in part, take the route through suppressing STAT3 pathway in Mia-PaCa2 pancreatic cancer cells. Other than STAT-3, NF-*κ*B also plays a vital role in development and metastasis of cancer and regulates expression of many genes including MMP-9 and FAK; hence we hypothesized that NF-*κ*B pathway would as well be impacted by OV. As expected, OV inhibited the phosphorylation of NF-*κ*B at all doses but only reduced the protein level of NF-*κ*B at 20 *μ*M (Figure 3(b)). Moreover, a remarkable suppression was observed in the phosphorylation level of I*κ*B and IKK *α*/*β*, but not in the total I*κ*B and IKK *α*/*β* protein. It indicated that OV inactivated NF-*κ*B activity by inhibiting phosphorylation of IKK *α*/*β* and I*κ*B. Taken together, OV showed the inhibitory effects on STAT-3 and NF-*κ*B cascade.

## 4. Discussion

Pancreatic cancer is the fourth leading cause of cancer death in the United States. Due to the lack of early detection methods and absence of effective biomarkers, patients are usually diagnosed at a late stage with a less than 5% 5-year survival rate. However, there is still no effective therapy available for treating this aggressive fiend. Although most chemotherapy regimens utilize gemcitabine as the clinical standard of care for pancreatic cancer, patients commonly have limited response to this therapy. Combination therapy and targeted therapies have also been overall quite disappointing. Thus, seeking more effective therapeutic interventions for pancreatic cancer is desperate.

Over the past 50 years, emerging evidences suggest that many natural products derived from plants, microbes, or marine or terrestrial origins exhibit beneficial effects toward the prevention of cancer [23, 24]. Ovatodiolide, a diterpenoid from a Chinese* herb*, exerts various biological activities including suppression of dendritic cell maturation and immunostimulatory activities [25], inhibition of cytopathic effects of HIV-1 infection [26], antioxidant capacities [27], and anti-inflammatory effects [10]. As long-term inflammation predisposes to cancer [28], recent researches investigated the anticancer activity of OV and reported an OV-induced cytotoxic effect to some human cancer cell lines via inducing cell cycle arrest and apoptosis [11, 29, 30]. Nevertheless, there is no reveal about the anticancer and antimetastatic potential of OV for aggressive pancreatic cancer.

Herein we demonstrated that OV exerted the anticancer potential in pancreatic cancer cell lines through inhibiting cell proliferation and inducing apoptosis and partly took a route via NF-*κ*B and STAT3 pathway. In the present study, the observed inactivation of NF-*κ*B was caused by inhibition of IKK *α*/*β* activation and the subsequent suppression of I*κ*B phosphorylation. Moreover, OV also had inhibitory effect on protein kinases responsible for phosphorylation of NF-*κ*B, thereby leading to decrease of phosphorylated NF-*κ*B protein. Intriguingly, a suppression of NF-*κ*B expression was observed at 20 *μ*M of OV suggesting that higher concentration of OV might not only inactivate the kinase for NF-*κ*B phosphorylation but also induce the degradation process of NF-*κ*B. In addition to NF-*κ*B, OV also showed suppressive influence on STAT3 phosphorylation (Tyr705, Ser727) and its upstream kinase, including P38, ERK 1/2, and AKT. Noteworthy, OV showed discriminative deactivation on AKT Ser473 and Thr308. It is known that phosphorylation at both Thr308 and Ser473 is required for full activity of AKT. However, two phosphorylated residues are located at distinct structural motifs of AKT [31]. Thr308 is located at the activation loop in the central kinase domain, while the Ser473 resided at the C-terminal hydrophobic motif. Therefore, the unique motif structures around the two key phosphorylation sites of AKT may fit into the docking surface provided by OV. In addition, AKT is phosphorylated at Thr308 by PI3Kdependent kinase 1 (PDK1) [32] while the kinase responsible for Ser473 phosphorylation is mTORC2 [33]. Whether OV regulated the upstream kinase of AKT to cause the differential phosphorylation at either residue is worthwhile for further investigation.

Cancer metastasis including steps of cell migration and invasion depends on dynamic changes in the expression of cell adhesion molecules and integrins, as well as the changes in the expression of extracellular proteases. MMPs belong to the zinc-metalloproteinases family and are involved in the breakdown of extracellular matrix in many normal physiological processes. It also has been shown that MMP-9 facilitates tumor invasion, metastasis, growth, and angiogenesis by degradation of collagen IV in basement membrane [34, 35]. In pancreatic cancer cell line, the poorly differentiated status of PANC-1 cells was associated with enhanced MMP activity [36]. Moreover, the MMP-9 expression in pancreatic ductal carcinoma was found to be correlated with lymph node involvement and occurrence of distant metastases [37]. In the present study, we demonstrated the antimetastatic effect of OV on Mia-PaCa2 cells in a dose-dependent manner by cell invasion assay. In addition, Mia-PaCa2 cells showed a significant decrease in MMP-9 mRNA expression when treated with OV. It has been reported that expression of MMP-9 is regulated by different transcriptional factors including NF-*κ*B [38, 39]. Overexpression of I*κ*B*α* in rabbit and human vascular smooth muscle cells suppressed the upregulation of MMP-9 [40], which indicated that the activation of NF-*κ*B is essential for MMP-9 expression. As we describe above, treatment of OV resulted in the inactivation of NF-*κ*B and its upstream regulators. Therefore, these data suggested that OV suppressed invasiveness of Mia-PaCa2 cells, at least in part, via inhibiting NF-*κ*B-mediated MMP-9 expression.

After reaching the secondary site, the metastatic cancer cells need to settle down by adhesion and then proliferate to form new tumor. Loss of adhesion ability may block the metastases formation; thus, cancer cell adhesion is also an important step in metastasis [20]. FAK is a focal adhesion-associated protein kinase which is important in cell-ECM-mediated signaling. It controls cellular adhesion, spreading processes, and transcriptional events promoting epithelial-to-mesenchymal transition (EMT) through kinase-dependent or kinase-independent fashions [41]. It was found that the strengthening rate was enhanced by FAK-regulated integrin activation and binding during the early stages of adhesion [42]. NF-*κ*B is a well-characterized transcription factor that activates the expression of FAK transcription [43]. Moreover, it has been reported that STAT3 participates in Eps8-induced FAK expression [44]. In our study, the cell adhesion ability, as well as FAK mRNA expression, significantly decreased after OV treatment. Western blot results also showed the inhibitory effect of OV on phosphorylation of NF-*κ*B, STAT3, and upstream regulators of STAT3 (P38, ERK1/2, and AKT Ser473). These results demonstrated the antimetastatic action of OV in blocking cell adhesion ability probably through inhibiting NF-*κ*B- and STAT3-mediated FAK transcription. It is worthy to note that, after exposure to extracellular pressure, AKT can regulate FAK through binding to FAK and inducing its phosphorylation which in turn promotes FAK autophosphorylation. Subsequently, FAK activates downstream signaling to regulate cancer cell adhesion [45]. Therefore, the inhibition of FAK-dependent cell adhesion induced by OV might partly contribute to the decreased amount of total or phosphorylated AKT protein.

**Figure 4:**
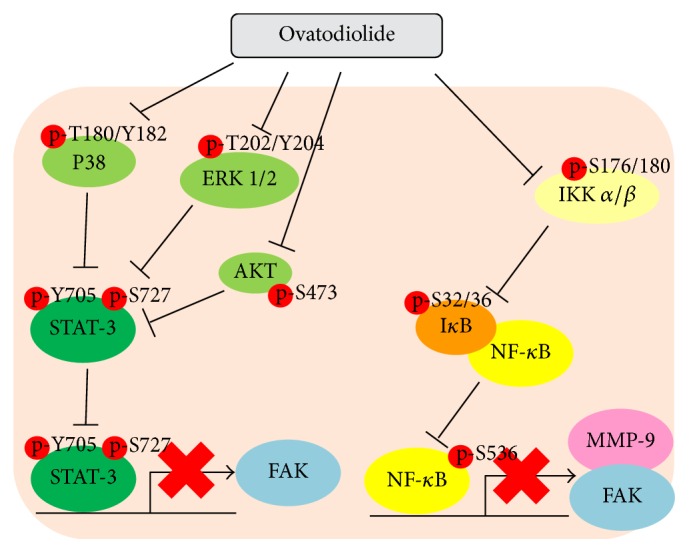
Schematic representation of OV-inhibited signaling cascades in Mia-PaCa2 cells.

In conclusion, we have demonstrated for the first time that OV could potentially inhibit Mia-PaCa2 pancreatic cancer cells proliferation and induce apoptosis through modulation of NF-*κ*B and STAT3 pathway. Moreover, the antimetastatic effect of OV on Mia-PaCa2 is not only by suppressing cell invasive ability but also by interfering with cell-matrix adhesion. Such effects might be due to the OV-induced inactivation of NF-*κ*B and STAT3 pathway and consequently abatement of MMP-9 and FAK transcription (Figure 4).

Up to now, there is no direct evidence about any predictable transporter or receptor on the membrane that OV interacts with. Nevertheless, OV is a small molecule (MW = 328) with low polarity that can diffuse through the lipid rich region of cell membrane readily. In addition, the log⁡*P* value of OV computed by PubChem is 2.4, which adumbrates the good cell permeability of this compound [46]. Taken together, our findings reveal a new therapeutic and antimetastatic potential of ovatodiolide for pancreatic cancer treatment.
